# Dendritic and Synaptic Degeneration in Pyramidal Neurons of the Sensorimotor Cortex in Neonatal Mice With Kaolin-Induced Hydrocephalus

**DOI:** 10.3389/fnana.2019.00038

**Published:** 2019-04-24

**Authors:** Omowumi M. Femi-Akinlosotu, Matthew T. Shokunbi, Thajasvarie Naicker

**Affiliations:** ^1^Department of Anatomy, College of Medicine, University of Ibadan, Ibadan, Nigeria; ^2^Department of Surgery, College of Medicine, University of Ibadan, Ibadan, Nigeria; ^3^Optics & Imaging Centre, School of Laboratory Medicine and Medical Sciences, College of Health Sciences, University of KwaZulu-Natal, Durban, South Africa

**Keywords:** neonatal hydrocephalus, sensorimotor cortex, golgi staining, pyramidal neurons, synaptophysin, dendrites, synapses

## Abstract

Obstructive hydrocephalus is a brain disorder in which the circulation of cerebrospinal fluid (CSF) is altered in a manner that causes expansion of fluid-filled intracranial compartments particularly the ventricles. The pyramidal neurons of the sensorimotor cortex are excitatory in nature and their dendritic spines are targets of excitatory synapses. This study evaluated the effect of hydrocephalus on dendritic arborization and synaptic structure of the pyramidal neurons of the sensorimotor cortex of neonatal hydrocephalic mice. Sterile kaolin suspension (0.01 ml of 250 mg/mL) was injected intracisternally into day old mice. Control animals mice received sham injections. Pups were weighed and sacrificed on postnatal days (PND) 7, 14 and 21. Fixed brain tissue blocks were silver impregnated using a modified Golgi staining technique and immunolabeled with synaptophysin to determine dendritic morphology and synaptic integrity respectively. Data were analyzed using ANOVA at *α*_0.05_. Golgi staining revealed diminished arborization of the basal dendrites and loss of dendritic spines in the pyramidal neurons of hydrocephalic mice. Compared to age-matched controls, there was a significant reduction in the percentage immunoreactivity of anti-synaptophysin in hydrocephalic mice on PND 7 (14.26 ± 1.91% vs. 62.57 ± 9.40%), PND 14 (4.19 ± 1.57% vs. 93.01 ± 1.66%) and PND 21 (17.55 ± 2.76% vs. 99.11 ± 0.63%) respectively. These alterations suggest impaired neuronal connections that are essential for the development of cortical circuits and may be the structural basis of the neurobehavioral deficits observed in neonatal hydrocephalus.

## Introduction

Hydrocephalus is a serious problem in sub-Saharan Africa (Salvador et al., 2014). It is an incapacitating disorder (Turgut et al., 2018) affecting 0.2–3.5/1,000 healthy births (Krause et al., 2018) and is the most common neurologic disorder requiring surgery in children (Vogel et al., 2012). The structural changes in the brain that accompany this disorder have been well described. Stretching of the ependymal layer, thinning of the corpus callosum, extracellular oedema and reduced cortical thickness have been observed to correlate with the degree of hydrocephalus (Olopade et al., 2012). Myelin sheath injury consists of attenuation, lamella separation and accumulation of myelin debris and focal degeneration as well as delayed myelination (Del Bigio et al., 1997; Castejón and de Castejón, 2004; Ayannuga et al., 2016). The changes in the sensorimotor cortex at the cellular and ultrastructural level have not been fully described.

A major element of the arrangement of the sensorimotor cortex is the morphology and deployment of pyramidal neurons which are the major source of intrinsic excitatory cortical synapses. The dendritic spines of these neurons are the main postsynaptic target of excitatory synapses (DeFelipe, 2015). Specializations in their structure are likely to influence cortical function at the subcellular, cellular and systems levels (Jacobs and Scheibel, 2002; Elston, 2003; Spruston, 2008; Luebke, 2017). More specifically, complexity in dendritic structure determines their biophysical properties thus influencing their functional capacity and potential for plastic changes (Elston, 2007; Spruston, 2008; Luebke, 2017). The functions of the sensorimotor cortex are therefore dependent on the connections and branching patterns of its pyramidal neurons.

Synapses develop concurrently and/or successively in different layers of the cerebral cortex. They establish the cell-to-cell communication which is paramount to the function of the cerebral cortex. Refinement of synaptic connections is a key step in the formation of neuronal circuits (Wang et al., 2011; Elston and Fujita, 2014). Cortical areas do not possess most of their defining characteristics until the post-natal period (Stocker and O’Leary, 2016). Synaptic vesicle membrane proteins participate in the storage of neurotransmitters, in the docking, fusion and recycling of synaptic vesicles (Grabs et al., 1994). Synaptic vesicles contain characteristic proteins, one of which is synaptophysin. It is located in the synaptic vesicles of membranes proteins and a constituent of the family of presumptive channel protein and the most abundant synaptic vesicle protein (Grabs et al., 1994). It is unclear how the hydrocephalic process influences the development of the synaptic landscape and the larger dendritic geometry in pyramidal neurons of the sensorimotor cortex. This study was designed to evaluate this relationship.

## Materials and Methods

**Figure 1:**
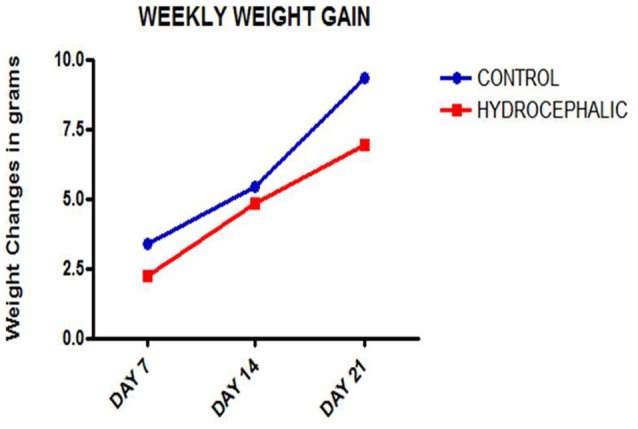
Line graph showing the mean weekly weight gain of the control and hydrocephalic mice. The hydrocephalic mice weigh significantly less than the control mice (*p < 0.05; ***p < 0.001).

The animals were handled with care in accordance with the protocol of Institutional Animal Care and Use Committee of the National Institute of Health, United States of America. Ethical approval for the study was obtained from the University of Ibadan Animal Care and Use Research Ethics Committee (UI-ACUREC/App/2014/008). Hydrocephalus was induced in day-old albino mice by intracisternal injection of 0.1 ml of 250 mg/ml sterile kaolin suspension. Control mice received a sham injection. The animals were weighed weekly and assessed for the development of hydrocephalus. These mice were sacrificed on postnatal days (PND) 7, 14 and 21 and were perfused transcardially first with normal saline for 3 min to wash out the blood cells and then for 15–20 min with 4% paraformaldehyde (PFA) until well fixed, using pallor of the liver and stiff muscles as an indication of good fixation. The brains were quickly dissected out and fixed for 4 h in the same solution. The brain samples were then rinsed, transferred into 0.1% sodium azide in 0.1 M phosphate buffered saline (PBS) and stored at 4°C until sectioning. The brain samples were bisected in coronal plane, at right angle to a horizontal tangent at the level of the optic chiasm and the proximal halves so obtained were examined grossly. For quantitative evaluation of synaptophysin positivity and estimates of dendritic lengths, we obtained samples from the dorsolateral area of the sensorimotor cortex as shown in Figure 1. This area contains the primary and secondary sensor cortices for the trunk and limbs.

### Hematoxylin and Eosin Staining

Four micron coronal sections were mounted on Leica X-tra adhesive precleaned micro-slides and stained with hematoxylin and eosin.

### Golgi Staining

Fresh brain tissues (sensorimotor cortex) were placed in 3% potassium bichromate for 5 days (avoiding the penetration of light by covering the specimen bottles with aluminum foil and placing in dark chamber). Tissues were placed in fresh solutions every day. The tissue blocks were transferred into 2% silver nitrate solution for 3 days at room temperature (avoiding light penetration and changing tissues into fresh solutions everyday). Before putting blocks into silver nitrate solution, a filter paper is used to absorb excess solution. Silver nitrate solution is changed several times until brown participates do not appear. Dehydrate tissues through 70%, 90%, 100% alcohol and xylene for 5 min each. Infiltrate the dehydrated tissues in molten wax at 56°C for 30 min. Tissues were then embedded in paraffin wax using molten wax dispenser Leica EG 1150H and then cooled over Leica EG 1150C. Microtome (Lieca RM 2135) cut sections (60 μm thick) were then mounted on Leica X-tra adhesive precleaned micro-slides (26 × 76 × 1.0 mm), air dried for 10 min and cover-slipped using DPX.

### Immunohistochemical (IHC) Staining

Immunohistochemical (IHC) Staining for synaptophysin was performed using EnVision™ FLEX staining kits (K8023/K8000). Tissue sections were cut at 4 μ with Lieca RM 2135 microtome floated onto Leica H1 1210 water bath at 50°C, and mounted on positively charged Leica X-tra adhesive pre-cleaned slides. The sections were dried on a hot plate (Sakura 1452; 230 V/60 Hz/ 160 VA) for 1 h at 60°C. The dried sections were oven baked (Memmert 400) at 60°C for 24 h. They were then processed through EnVision™ FLEX Target Retrieval Solution, High pH (50×) or Low pH (×50) diluted 1:50 with dH_2_O, EnVision™ FLEX Wash Buffer (20×) diluted 1:20 with dH_2_O and EnVision™ FLEX DAB + Chromogen and Substrate Buffer diluted 1:20 with substrate buffer [1 drop (50 μl) of DAB + Chromogen per 1 ml of substrate buffer]. The Ready-To-Use antibody (synaptophysin) was mixed as equal part of primary antibody and HRP (incubated for 15 min) in an amount required for all slides. Mouse serum was added to the above mixture in a ratio of 1:50 (incubated for 5 min). The sections were rinsed in copious amount of tap water, counterstained with hematoxylin, dehydrated in ethanol (95% and 100%), cleared in xylene and cover-slip with DPX.

### Data Acquisition

Image stacks of the golgi fixed brain sections were obtained using Leica application suite version 3.3.0 (Switzerland). Two-dimensional image stacks having 640 pixels × 480 pixels frame size at 40× magnification were used in recording the basal dendrites. To ensure a homogenous pyramidal neuronal population, the neurons selected for analysis fulfilled the following criteria: (a) the cell body and dendrites were completely impregnated; (b) the neurons were isolated from the surrounding neurons; and (c) all the dendrites were visible within the plane of focus (Xiong et al., 2013). We calculated the basal dendrite length as a mean of 10 basal dendrites per animal in the layer V of the sensorimotor cortex by using ImageJ analysis software version 1.49.

The brain sections processed for immunohistochemistry were viewed under Zeiss Axio Scope A1 microscope version 4.8.2.3 (Carl Zeiss Microscopy GmbH). Percentage immunoreactivity was determined using the ImageJ software at 40× magnification. The impact of hydrocephalic process was evaluated by comparing the percentage immunoreactivity of synaptophysin in hydrocephalic animals and controls.

### Statistical Analysis

All data were presented as means ± standard error of mean (SEM) and compared across the groups using the GraphPad Prism version 7 for Windows Software (San Diego, CA, USA) with confidence interval calculated at 95%.

## Results

### Body Weight

**Figure 2:**
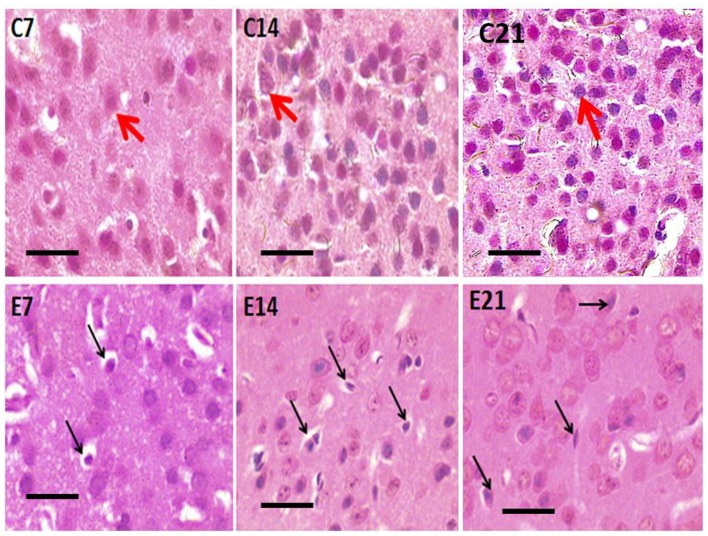
Photomicrograph of H&E stained sensorimotor cortex of control mice (C7, C14, C21) showing normal pyramidal neurons (red arrows) and hydrocephalic mice (E7, E14, E21) showing shrunken pyramidal neurons (black arrows). Scale bar: 10 μm.

A comparison of the weekly weight gain of the control and the hydrocephalic groups revealed a statistically significant reduction in the hydrocephalic groups especially on PND 7 and 14 (Figure 2 and Table 1). In this study, both control and experimental animals gained weight with age. However, the hydrocephalic pups had significantly lower body weights compared to their age-matched controls. The hydrocephalic mice decreased in weight in the 1st week of hydrocephalus induction, increased in weight by the 2nd week (at par with the age-matched controls) but lost weight again in the 3rd week post induction of hydrocephalus.

**Table 1:** Body weights of mice following kaolin injection showing Mean ± SEM in grams (** *p* < 0.01, ***p < 0.001 in comparison to controls; *n* = 20).

Days post induction	Control *n* = 20	Hydrocephalic *n* = 20
7	3.45 ± 0.06	2.39 ± 0.11**
14	5.10 ± 0.14	5.44 ± 0.28
21	8.82 ± 0.21	6.26 ± 0.27***

### Hematoxylin and Eosin Staining

**Figure 3:**
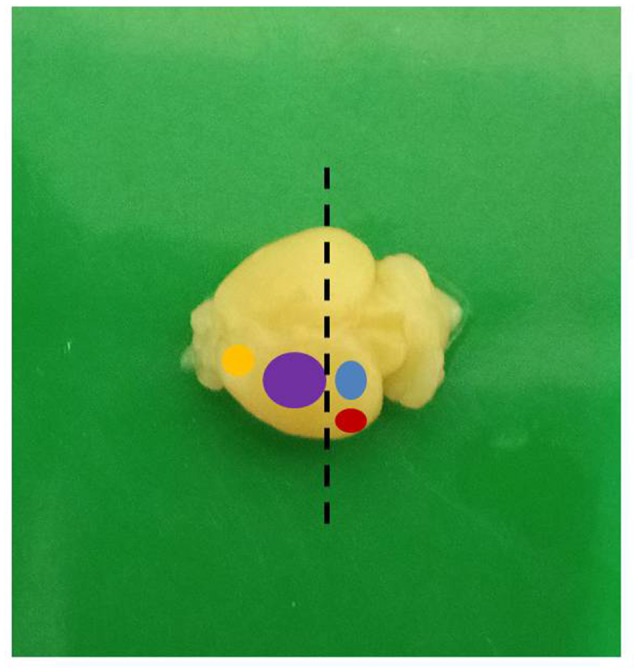
A mouse brain showing the different functional areas (yellow: motor; purple: sensorimotor; blue: visual; red: auditory; dashed line: level of sectioning).

Abnormal neurons were observed in the sensorimotor cortex of all the hydrocephalic mice groups—7, 14 and 21 days post-induction when compared with the control cortex (Figure 3). The neuropil of the control mice showed distinct pyramidal neurons with scanty pyknosis while the hydrocephalic neuropil revealed numerous pyknotic neurons. These dying/dead neurons characteristically resemble “open-faced” nuclei (highly reactive cells that are responding to a pathological state).

### Golgi Staining

The dendrites of cortical excitatory neurons are characterized by their spines. The Golgi impregnation technique allowed clear visualization of the arborization of the apical and basal dendrites as well as their spines. The dendrites of the pyramidal neurons in the control mice were long, arborized extensively and intertwined with those of neighboring neurons. In the hydrocephalic groups, the dendrites were very short, and arborization was scanty. There were little or no basal dendrites in the field of display of these experimental groups.

**Figure 4:**
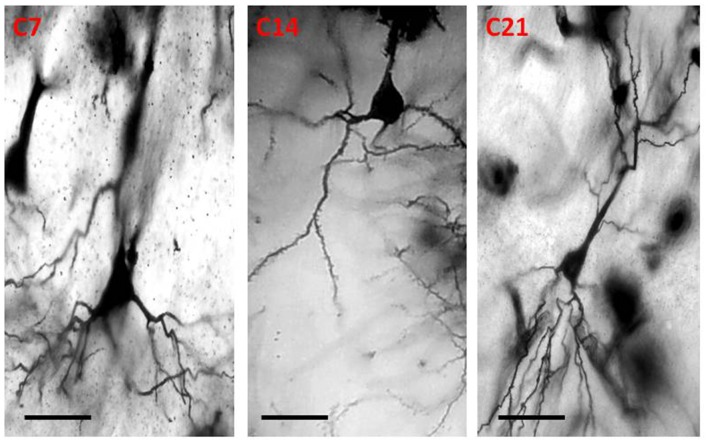
Photomicrograph of Golgi staining of the sensorimotor cortex of control mice on post-natal days 7, 14, 21 showing the dendritic arborization of the pyramidal neurons (C, Control mice). Scale bar: 20 μm.

**Figure 5:**
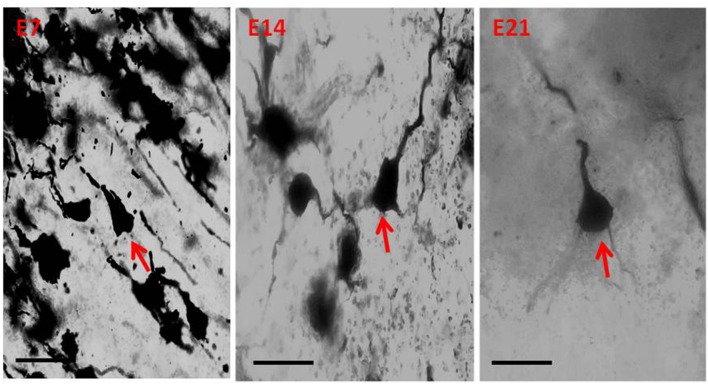
Photomicrograph of Golgi staining of the sensorimotor cortex of the hydrocephalic mice on post-natal days 7, 14, 21 showing loss of dendritic arborization and absence of basal dendrites (red arrows) of the pyramidal neurons (E, Experimental hydrocephalic mice). Scale bar: 20 μm.

Qualitative assessment of the sensorimotor cortex of the control mice showed an abundance of both apical and basal dendrites accounting for their prominent arborization (Figure 4). Compared to their age-matched controls, the impregnation of the sensorimotor cortices in the hydrocephalic mice revealed three distinct features: (a) markedly smaller arborization of the dendrites (both apical and basal dendrites); (b) fewer basal dendrites; and (c) fewer dendritic spines (Figure 5).

**Figure 6:**
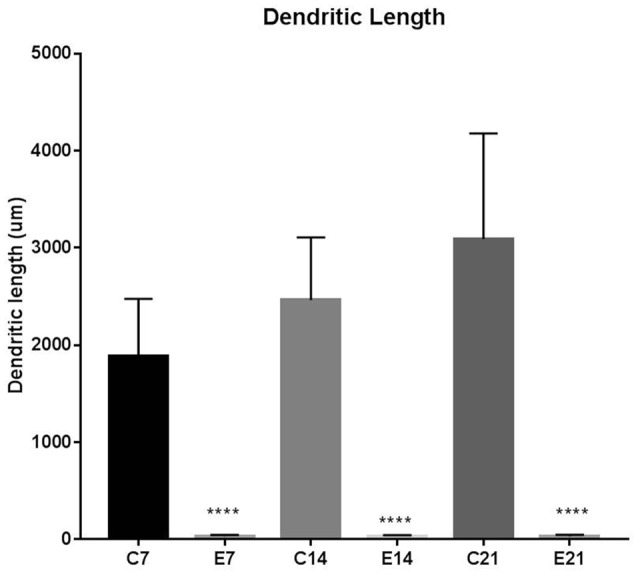
Bars represent the mean ± SEM of basal dendrite length (C, Control; E, Experimental; ****p < 0.001; *n* = 20).

The impression obtained in the qualitative evaluation described above is supported by measurements which revealed that the mean (±SEM) length of the basal dendrites was significantly reduced in the hydrocephalic groups on PND 7 (32.09 μm ± 4.17), PND 14 (33.00 μm ± 3.63) and PND 21 (32.38 μm ± 4.84) compared to length in their age matched controls (1889 μm ± 186.70, 2466 μm ± 202.80 and 3091 μm ± 343.60, respectively; Figure 6).

### Synaptophysin Immunohistochemistry

**Figure 7:**
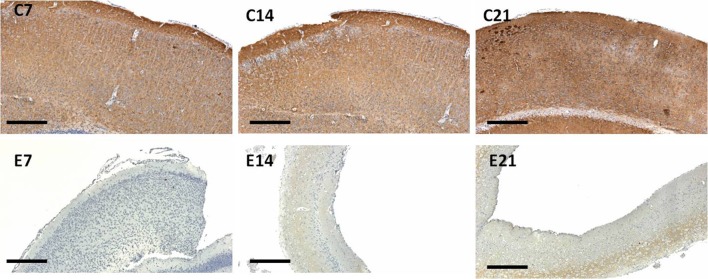
Immunoreactivity of synaptophysin in the sensorimotor cortex of neonatal control and hydrocephalic mice showing different levels of positivity (C7, Control postnatal day 7; C14, Control postnatal day 14; C21, Control postnatal day 21; E7, Experimental postnatal day 7; E14, Experimental postnatal day 14; E21, Experimental postnatal day 21). Scale bar: 100 μm.

**Figure 8:**
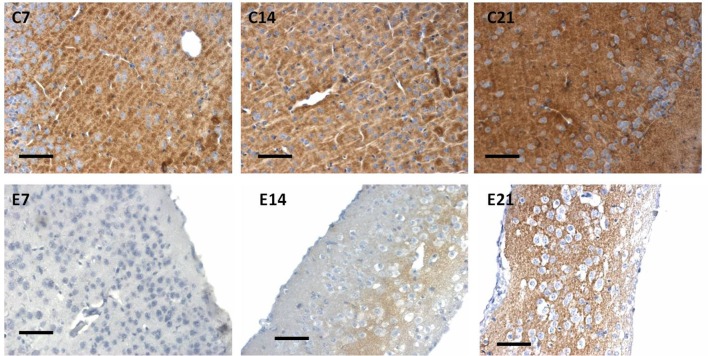
Immunoreactivity of synaptophysin of control and hydrocephalic mice showing a reduction in immunoreactivity in the hydrocephalic group. Note the vacuolation and the little number of the cells in E14 and E21compared to the compact arrangement of the cells in their age-matched control group (C21: Control postnatal day 21; E21: Experimental postnatal day 21). Scale bar: 20 μm.

Little information is available on the expression of the synaptic protein, synaptophysin in neonatal hydrocephalic sensorimotor cortex over time despite the fact it is thought to be present in virtually in every synapse of the brain. Therefore, synaptophysin immunoreactivity was performed to ascertain its presence in the neonatal hydrocephalic state. The immunoreactivity of synaptophysin in the control animals revealed a positive reaction with an increase in the intensity as the mice progress in age, however, the expression of synaptophysin immunoreactivity was reduced in the hydrocephalic groups (Figure 7). The laminar architecture of the hydrocephalic groups was distorted and the neuropil appears spongy with the presence of a lot of vacuoles (Figure 8).

The intensity of the synaptophysin immunoreactivity in the sensorimotor cortex of the control mice indicates that the cell bodies, axons and apical dendrites of the pyramidal neurons which were numerous as seen in the hematoxylin and eosin staining were all stained. Moreover, the immunoreactivity of the synaptophysin increases as the age of the mice increased (Figures 7, 8: C7, C14, C21). In the cortices of the hydrocephalic rats which had fewer pyramidal neurons and arborizations (golgi staining), the synaptophysin immunoreactivity was reduced. It was also observed that the intensity of the synaptic protein gradually increased over time in the hydrocephalic cortices, but the change was not as pronounced in the control mice (Figures 7, 8: E7, E14, E21). The neuropil of the control mice appeared compact and regular, whereas it was loose and spongiform in hydrocephalic mice, especially by E14 and E21 (Figure 8).

The percentage positivity of synaptophysin showed significant reduction in the percentage immunoreactivity in PND 7 (14.26% ± 1.91), PND 14 (4.19% ± 1.57) and PND 21 (17.55% ± 2.76) compared to controls 62.57% ± 9.40, 93.01% ± 1.66 and 99.11% ± 0.63, respectively (Table 2) which further accentuated the former observations. Altogether, synaptophysin immunoreactivity was greatly affected by the hydrocephalic insult.

**Table 2:** Percentage positivity of synaptophysin immunoreactivity between the control and experimental mice showing Mean ± SEM (***p < 0.001; *n* = 20; SEM, standard error of mean).

Post natal day (PND)	Sham control mice	Hydrocephalic mice
7	62.57 ± 9.40	4.19 ± 1.57***
14	93.01 ± 1.66	14.26 ± 1.91***
21	99.11 ± 0.63	17.55 ± 2.76***

## Discussion

In this study, we evaluated the impact of hydrocephalus on the microcircuitry of the sensorimotor cortex of the neonatal mouse by examining its laminar architecture, and the morphology, dendritic, and synaptic architecture of its principal neuron, the pyramidal neuron. We found that hydrocephalus adversely affected the neonatal body weight, the morphology of the pyramidal neurons and their dendritic arborization.

Both the control and hydrocephalic pups gained weight with age, but the hydrocephalic animals were lighter in weight at the three points of measurement after birth and showed a smaller gradient of weight gain. The possible cause of this difference may be due to the neglect of the hydrocephalic neonates by their mothers, neurogenic muscle atrophy, lethargy and general weakness of the hydrocephalic pups. Reduction in weight has been reported to be one of the first signs of development of hydrocephalus in experimental models (Del Bigio, 2001; López et al., 2003; McMullen et al., 2012; Johnston et al., 2013; Shaolin et al., 2015; Olopade et al., 2012). The body weight is an important index in developmental biology in neonatal pups and may be a pointer to an on-going pathologic condition.

Our study revealed a reduction of dendritic branching and arborization of the pyramidal neurons in hydrocephalic pups. The golgi stained sections of the hydrocephalic mice showed a reduction in the dendritic spines of both the apical and basal dendrites. Alterations in the morphology and arborization pattern of dendrites, the morphology and density of their spines have functional significance. Spines and spine densities are malleable structural configurations which reflect changes in development and in disease of the brain (Sakić et al., 1998). Several studies have reported dendritic alterations in hydrocephalus thus McAllister et al. (1985) observed varicosities and reduction in the spines of the basal dendrites of hydrocephalic newborn rats, Kriebel et al. (1993) found that dendritic appendage morphology was altered in hydrocephalic kittens while Harris et al. (1996) reported a reduction of both apical and basal dendritic length in hydrocephalic rats by 49%–57% compared to the controls. Behavior related dendritic changes have been observed in the sensorimotor parietal cortex (Lolova, 1989; Kolb and Gibb, 1991). The main receivers of excitatory inputs are the dendritic spines and they are essential in managing the signal input and output of the neurons. Evidence suggests that the apical and basal dendritic spines show unique and differential susceptibility (Xiong et al., 2013). Chakraborti et al. (2012) documented that the irradiation of the cranium of young adult mice led to a reduction in the density of the spines of basal dendrites but not those of the apical dendrites of the pyramidal neurons of Cornus Ammonis 1 of the hippocampus. A similar apico-basal differential was observed for dendritic spines of aging C57BL/6 mice (von Bohlen Und Halbach, 2006). The analyses of the somatic and the dendritic anatomy of Golgi stained pyramidal neurons of the sensorimotor cortex of these neonatal hydrocephalic mice revealed differences between the control and the experimental groups. Some of these differences are loss of basal dendrites and spines, reduced dendritic arborization, disintegration of apical dendrites, the reduction in the quality of dendrite processes, variations of dendritic spines and spine denudation of some dendritic segments. The differences observed could be as a result of increased pressure, alterations in the vessels, white matter injury and de-afferentation (McAllister et al., 1985). Most of the extrinsic and intrinsic afferents of the cortex end on the spines of the dendrites of pyramidal neurons (Peters and Kaiserman-Abramof, 1970), therefore any change in the quality of their branching pattern or morphology could have significant functional importance. Huttenlocher (1974) documented remarkably fewer arborization of the dendrites of pyramidal neuron in the neocortex of mentally impaired children. Stunted growth of the dendrites at both the apex and base and their damage were also described. In this study, the basal dendrites seem to be more severely affected than the apical dendrites while the dendritic branches were reduced. Studies in neonates with abiogenic neurobehavioral impairment have revealed dendritic alterations (Purpura, 1974, 1975; Purpura et al., 1982). Fetal alcohol syndrome in infants which is often associated with mental impairment is also associated with varying degrees of degenerations of dendritic branch patterns (Stoltenburg-Didinger and Spohr, 1983). The loss of basal dendrites in this study corroborates what was observed by McAllister et al. (1985).

McAllister et al. (1985) also revealed that the pyramidal neurons of the cerebral cortex have reduced number and dimensions of dendrites between the 10th and 12th post-natal days. Networks of synapses are essential for the learning and the memory in the brain and are characteristically being fabricated and removed during growth and in answer to impulses. Quantification of the alterations in the entire synaptic density is a crucial requirement for understanding the networking of these structures in diseased conditions (Navlakha et al., 2013). Harris et al. (1997) observed a decrease in the dimensions of the branching mosaic of dendrites in both the apex and the base of the dendrites; the distance measured from the body of the dendrite was diminished in the hydrocephalic rats by 50%–57%, while the total length of the dendritic arbors was reduced between 61% and 77%. In this study, the basal dendrites were greatly affected when compared to the apical dendrites; this is in agreement with what was observed by Harris et al. (1996). Changes in the morphologic pattern are noteworthy because researches have suggested that the apical and the basal dendritic pattern might be the domain of distinct classes of inputs and the spines of the dendrites might be exponentially stress-susceptible (Kang et al., 2015). The spines of dendrites are the principal domains of excitatory glutamatergic input to pyramidal neurons of the cerebral cortex. The spines play vital roles in the biochemical chambers (Yuste et al., 2000) and they add energy to the tree of the dendrite (Kang et al., 2015). These pathological changes in the cerebral cortex of human hydrocephalus patients are considered to result from an initial mechanical injury because of the high cerebrospinal fluid (CSF) pressure, followed by secondary changes associated with increased interstitial oedema, ischemia, and oxidative stress (Zhang et al., 2017).

Synaptophysin, a synaptic vesicle membrane protein ubiquitous in the central nervous system, is one of the commonest targets for immune histochemical detection of synapses. Although its precise function is unknown, it appears early and in abundance in growing synapses. Studies have shown that showed that this protein is not only present in the full-grown end points but also in out-growing axons (Scarfone et al., 1991; Bergmann et al., 1993; Ovtscharoff et al., 1993). In this study, neonatal hydrocephalus was associated with a significant reduction in the expression of the synaptophysin immunostaining. This is consistent with the reduction of the dendritic processes as shown by Golgi staining and supports the notion that impaired dendritic branching and spine formation can result from deficits in synaptogenesis in hydrocephalic brains (Del Bigio, 2010). Although immunoreactivity was delayed in appearance and also diminished in the hydrocephalic brains compared to control, staining increased with age and was more intense in the deeper layers of the cortex. The decreased synaptophysin reactivity in the experimental mice is also suggestive of synaptic pathology (Eastwood et al., 2000), which might take place as the process of deterioration of tissue continues (Tanaka et al., 2000), may mirror deformities of either post-transcription of synaptophysin and decrease in the number of axonal connections of cerebral cortical neurons (Rahmy and Hassona, 2004).

The releases of neurotransmitter at the synapses of neurons are mediated by the synaptic vesicle through fusion and exocytosis at proliferative zones (Xian et al., 2009). The mosaic of immunoreactivity revealed by synaptophysin marker in the sensorimotor cortex of the hydrocephalic pups concurs with earlier documentations of developmental alterations in the cortical synaptic density in the cortex of the cerebrum (Rakic et al., 1986; Bourgeois and Rakic, 1993; Glantz et al., 2007). Modified synaptic removal in the pathogenesis of hydrocephalus has since been predicated. The easiest deduction of these outcomes is that they show the design of synapses growth over the post-natal sensorimotor cerebral cortex growth, which is in agreement with the results obtained by Glantz et al. (2007). Furthermore, the magnitudes of proteins in the synapses might partly show the feature of the synapses (Eastwood and Harrison, 2001). Nakamura et al. (1999) observed that when synapses are pruned early in post-natal development, the decline in number of synapses is preceded by loss of vesicles within the synapses. All these normal developmental achievements could have been decreased by the process of hydrocephalus in the mice neonatal brain. The functional differences between cerebral cortical areas might be mirrored by both the density and number of synaptic morphologies (Elston et al., 2011).

It has been documented that reduced state of synaptophysin also lowers the function and transmission of pre-synaptic sites (Pinto et al., 2013). Two synaptic vesicle proteins i.e., synaptophysin and synaptobrevin mingle together to manage the renascence and endocytosis of the synaptic vesicle. The full growth and development of pre-synaptic communication is needed for the transfer from a quiescent to a clamorous synapse revealing that adequate degrees of synaptophysin is required to manage vesicle exocytosis and endocytosis to maintain the activities of the neural network (Pinto et al., 2013). Synaptophysin is therefore needed to maintain the location and effectiveness of pre-synaptic terminals. There is a likelihood that the fast growing equilibrium between these pre-synaptic proteins manages the initial stability of pre-synaptic activity and underpins diversities in the full growth of pre-synaptic activities in the sensorimotor regions. IHC studies have demonstrated altered synaptic vesicle proteins which may reflect in a loss of synaptic contact (Del Bigio and Zhang, 1998; Del Bigio, 2004). Histochemical and biochemical analyses also showed that the composition and by inference the function of neurons are altered in an hydrocephalic state (Del Bigio and Vriend, 1998; Kondziella et al., 2002; Del Bigio, 2004).

Discrepancies in the development and expression of the pre-synaptic protein and synaptophysin may establish an easy foundation to further appreciate the growth of cortical circuitry. Infants with hydrocephalus are challenged in both their motor and executive functions (Krause et al., 2018) therefore understanding the changes in the dendritic arborization of the pyramidal neurons of the sensorimotor cortex of infant hydrocephalic mice may lead to pharmacological management that could be fabricated to target unique and vital neural mechanisms to debilitate neuronal remodeling and consequential behavioral damages. Knowledge of the mosaic of the normal growth of the cerebral cortical synapses is therefore crucial to a better knowledge of the various ailments that are synaptic malformation related.

## Ethics Statement

This study was carried out in accordance with the recommendations and approval of Animal Care and Use Research Ethics Committee (UI-ACUREC) guidelines of the University of Ibadan with the reference approval number UI-ACUREC/App/2014/008.

## Author Contributions

MS conceptualized and designed the project, supervised its execution, read, revised and approved the manuscript for submission. TN supervised the immunohistochemistry. OF-A performed the experiments, collected the data, performed the statistical analysis and wrote the first draft of the manuscript.

## Conflict of Interest Statement

The authors declare that the research was conducted in the absence of any commercial or financial relationships that could be construed as a potential conflict of interest.
